# Anomalous AMPK-regulated angiotensin AT_1_R expression and SIRT1-mediated mitochondrial biogenesis at RVLM in hypertension programming of offspring to maternal high fructose exposure

**DOI:** 10.1186/s12929-020-00660-z

**Published:** 2020-05-23

**Authors:** Yung-Mei Chao, Kay L. H. Wu, Pei-Chia Tsai, You-Lin Tain, Steve Leu, Wei-Chia Lee, Julie Y. H. Chan

**Affiliations:** 1 Institute for Translational Research in Biomedicine, Kaohsiung Chang Gung Memorial Hospital, Kaohsiung, 83301 Taiwan; 2 Department of Pediatrics, Kaohsiung Chang Gung Memorial Hospital and Chang Gung Univeristy College of Medicine, Kaohsiung, 83301 Taiwan; 3 Division of Urology, Kaohsiung Chang Gung Memorial Hospital, Kaohsiung, 83301 Taiwan

**Keywords:** Maternal high fructose, AMP-activated protein kinase, Sirtuins, Angiotensin type 1 receptor, Rostral ventrolateral medulla, Programmed hypertension

## Abstract

**Background:**

Tissue oxidative stress, sympathetic activation and nutrient sensing signals are closely related to adult hypertension of fetal origin, although their interactions in hypertension programming remain unclear. Based on a maternal high-fructose diet (HFD) model of programmed hypertension, we tested the hypothesis that dysfunction of AMP-activated protein kinase (AMPK)-regulated angiotensin type 1 receptor (AT_1_R) expression and sirtuin1 (SIRT1)-dependent mitochondrial biogenesis contribute to tissue oxidative stress and sympathoexcitation in programmed hypertension of young offspring.

**Methods:**

Pregnant female rats were randomly assigned to receive normal diet (ND) or HFD (60% fructose) chow during pregnancy and lactation. Both ND and HFD offspring returned to ND chow after weaning, and blood pressure (BP) was monitored from age 6 to 12 weeks. At age of 8 weeks, ND and HFD offspring received oral administration of simvastatin or metformin; or brain microinfusion of losartan. BP was monitored under conscious condition by the tail-cuff method. Nutrient sensing molecules, AT_1_R, subunits of NADPH oxidase, mitochondrial biogenesis markers in rostral ventrolateral medulla (RVLM) were measured by Western blot analyses. RVLM oxidative stress was measured by fluorescent probe dihydroethidium and lipid peroxidation by malondialdehyde assay. Mitochondrial DNA copy number was determined by quantitative real-time polymerase chain reaction.

**Results:**

Increased systolic BP, plasma norepinephrine level and sympathetic vasomotor activity were exhibited by young HFD offspring. Reactive oxygen species (ROS) level was also elevated in RVLM where sympathetic premotor neurons reside, alongside augmented protein expressions of AT_1_R and pg91^phox^ subunit of NADPH oxidase, decrease in superoxide dismutase 2; and suppression of transcription factors for mitochondrial biogenesis, peroxisome proliferator-activated receptor γ co-activator α (PGC-1α) and mitochondrial transcription factor A (TFAM). Maternal HFD also attenuated AMPK phosphorylation and protein expression of SIRT1 in RVLM of young offspring. Oral administration of a HMG-CoA reductase inhibitor, simvastatin, or an AMPK activator, metformin, to young HFD offspring reversed maternal HFD-programmed increase in AT_1_R and decreases in SIRT1, PGC-1α and TFAM; alleviated ROS production in RVLM, and attenuated sympathoexcitation and hypertension.

**Conclusion:**

Dysfunction of AMPK-regulated AT_1_R expression and SIRT1-mediated mitochondrial biogenesis may contribute to tissue oxidative stress in RVLM, which in turn primes increases of sympathetic vasomotor activity and BP in young offspring programmed by excessive maternal fructose consumption.

## Background

Emerging evidence from human and animal studies indicates that the increased risk for a number of cardio-metabolic diseases, including hypertension, diabetes, heart failure, myocardial infarction and chronic kidney disease, may have their origins from birth [1, 2]. According to the concept of developmental original of adult health and disease (DOHaD) [3], susceptibility to the development of those non-communicable diseases in adult life could be primed by exposures such as malnutrition, prenatal hypoxia, hyperglycemia, toxin and medication during fetal life [4]. In particular, elevated blood pressure (BP), a major determinant of morbidity and mortality burden to cardio-metabolic risk [5], can be programmed by maternal overnutrition [6, 7]. In a rodent model of DOHaD, we [8, 9] and others [10, 11] demonstrated that maternal exposure to a high fructose diet (HFD) during gestation and/or lactation programs the development of hypertension in adult offspring. Both vascular and renal dysfunctions have been proposed as the culprits [12, 13], with enhanced reactive oxygen species (ROS) and reduced nitric oxide (NO) availability as the major protagonists [14, 15].

Programmed hypertension of fetal origin is associated with a significant activation of the sympathetic nervous system (SNS) [15, 16]. In the rostral ventrolateral medulla (RVLM), where the sympathetic premotor cells reside [17], the bulk of evidence supports the argument that tissue oxidative [18] and nitrosative [18, 19] stress play pivotal roles in central mechanisms of sympathoexcitation associated with hypertension. At the molecular level, angiotensin type 1 receptor (AT_1_R)-dependent activation of the nicotinamide adenine dinucleotide diphosphate oxidase (NADPH oxidase) signaling [18–20], suppression of endogenous antioxidants [18, 19, 21], impairment of mitochondrial biogenesis [18, 19, 22] and changes in protein expressions of NO synthase (NOS) isoforms [19, 23] have all been reported to contribute to the pathogenesis of hypertension via sympathoexcitation.

AMP-activated protein kinase (AMPK), a nutrient sensing molecule known for its function to ‘fuel gauge’ cellular energy status [24], plays a key role in the regulation of whole-body energy balance [25]. For example, metabolic disturbances in the liver programmed by fetal malnutrition are mediated, at least in part, by the suppression of AMPK phosphorylation at the Thr^172^ residue [26]. More recently, an interplay between oxidative stress and nutrient sensing signals, including AMPK and sirtuin (SIRT), has been stipulated to be a major component in cardio-metabolic programming of fetal origin [27], although detailed underlying mechanisms of this interplay is wanting.

The present study was undertaken to interrogate whether maternal HFD contributes to the programming of sympathoexcitation and hypertension in adult life by altering the tissue ROS and/or NO homeostasis in RVLM of young offspring. We also delineated the molecular mechanisms underpinning the programmed dysfunctions in RVLM of HFD offspring. Our data revealed that maternal HFD induces tissue oxidative stress in RVLM that primes an increase in sympathetic vasomotor activity and BP in young offspring. We further identified that AMPK-regulated increase in AT_1_R expression and SIRT1-mediated dysfunction of mitochondrial biogenesis as potential mediators for the enhanced ROS production in RVLM of HFD offspring.

## Methods

### Animals

Virgin female (*n* = 24) and male (*n* = 12) normotensive Sprague-Dawley (SD) rats at age of 10 weeks purchased from BioLASCO (Taipei, Taiwan) were used for breeding. Animals were allowed to acclimatize in a temperature- (22 ± 1 °C), humidity- (55 ± 5%) and light- (12:12 light-dark cycle, light on from 08:00) controlled room in an AAALAC-International accredited animal facility for at least 14 days before the experiments. All experiments were approved by our institutional animal care and use committee (2,012,102,701 and 2,015,122,217), and were carried out in accordance to the guidelines for animal experimentation endorsed by that committee and conformed with the National Institutes of Health Guide for the Care and Use of Laboratory Animals.

### Measurement of blood pressure

We routinely measured systolic blood pressure (SBP) at 14:00–16:00 in rats under conscious condition using the noninvasive tail-cuff method based on electrosphygmomanometry (MK-2000; Muromachi Kikai Co., Tokyo, Japan). Offspring to dams fed maternal normal diet (ND) or 60% high fructose diet (HFD) chow were handled repeatedly and allowed to adapt to the restraint chamber for at least 3 days before the commencement of actual measurements. BP determinations were considered valid only when five consecutive readings were recorded from rats under resting condition and the values did not differ by more than 5 mmHg; the mean of the five readings was then recorded as the measured value. SBP was measured in ND and HFD offspring at age of 6, 8, 10 and 12 weeks; in some offspring until age of 18 weeks. We have previously validated [28, 29] and again in pilot experiment of the current study that BP obtained by tail-cuff plethysmography was comparable to those measured by radiotelemetry.

### Evaluation of sympathetic vasomotor tone

Animals were anesthetized with isoflurane (5% for induction and 2% for maintenance) via an anesthesia mask for cannulation of femoral artery. Satisfactory anesthesia was maintained by the absence of withdrawal reflex to hind paw pinch. Animals were allowed to breathe spontaneously with room air, and body temperature was maintained at 37 °C by a heating pad. The arterial pressure was monitored from the femoral artery cannula for 30 min between 14:00 and 16:00. The pressure signal was analyzed by an arterial blood pressure analyzer (Notocord, Le Pecq, France) to obtain systemic arterial pressure (SAP). Continuous on-line, real-time spectral analysis of SAP signals based on fast Fourier transform was used to detect temporal fluctuations in the low frequency (LF, 0.25–0.8 Hz) component of SBP signals. Power density of LF band was used as our experimental index to reflect sympathetic vasomotor tone [30, 31].

### Measurement of plasma norepinephrine concentration

Plasma norepinephrine (NE) level was measured by the o-phthaldehyde (OPA) method using high performance liquid chromatography with fluorescence detection [32]. In brief, 500 μL ice cold trichloroacetic acid was added to 500 μL plasma sample and mixed by vortexing for 20 min, followed by centrifugation at 15,000×*g* for 20 min at room temperature. The supernatant was filtered through a 0.22 μm syringe filter (Chroma Technology Corp., Bellows Falls, VT), mixed with 4 fold methanol and centrifuged at 12,000×*g* for 15 min at room temperature. The supernatant was filtered again and kept at − 80 °C until analysis.

The sample or standard NE solution (at concentrations of 1–50 mM) was mixed with the derivatization reagent OPA solution at 4:1 (V/V) and placed in dark for 15 min. Each 20 μL aliquot was injected into the HPLC system using a Rheodyne (model 7125, Merck KGaA, Darmstadt, Germany) injector. The HPLC system (Hitachi CM5000, Hitachi Corp., Tokyo, Japan) is comprised of a 5110 syringe pump system, a 5210 autosampler and a 5440 FL spectrofluorimetric detector. Chromatographic separation was achieved on a ZORBAX SB-C_18_ column (4.6 mm × 250 mm, 5 μm; Agilent Technologies, Taipei, Taiwan). The column temperature was maintained at 30 °C and the flow rate was 1 mL/min. Methanol and acetate buffer (20 mM, pH 3.5, with 1 mM Na_2_EDTA) with a ratio of 5:4 was used as the mobile phase, and the samples were eluted within 20 min. The fluorescence signal was detected with excitation and emission wavelengths of 340 nm and 450 nm, respectively. Retention time for NE was 2.2–2.6 min. The concentration of NE was computed by comparing the area under curve of each sample against standard solutions of known concentrations. Each sample was analyzed in triplicates and the results are shown as the mean of the three values.

### Measurement of blood biochemistry

All measurements were carried out in ND and HFD offspring at age of 6, 9 and 12 weeks, and each assay was performed in triplicates. The animals were fasted for at least 15 h before blood samples were collected from a punch at the tip of the tail. Glucose levels were analyzed using a glucose oxidase kit (Roche, Basel, Switzerland) according to the manufacturer’s instructions. For plasma insulin measurement, 200 μL blood sample was centrifuged at 2000×*g* for 15 min. Fasting plasma insulin was analyzed by means of an ELISA kit (Mercodia, Uppsala, Sweden). Samples were first interacted with plate-coated mouse monoclonal anti-insulin, followed by reacting with peroxidase-conjugated anti-insulin antibodies. The bound conjugate was detected by reaction with 3,3′,5,5′-tetramethylbenzidine and read by a spectrophotometer (Thermo Fisher Scientific Inc., Waltham, MA) at 450 nm after the reaction was stopped by adding stop solution. The detected insulin and glucose concentrations were then used to calculate the ‘homeostasis model assessment’ (HOMA) indices of insulin resistance (IR) based on formulas reported previously [33].

The concentration of fasting plasma triglycerides was detected by a triglycerides assay kit (Randox, Antrim, UK). Ten μl plasma from fasting animals was incubated with the reaction mixture at 25 °C for 20 min. Triglyceride concentration was detected by a spectrophotometer (Thermo Fisher Scientific Inc.) at 570 nm. The concentration of fasting plasma leptin was detected using a rat leptin quantikine ELISA kit (R&D Systems, Minneapolis, MN). In brief, after 10 fold dilution into calibrator diluent, 50 μl solution was used to react with equal volume of acetic acid (2.5 N) and urea (10 M) according to the protocols provided by the manufacturer. The level of plasma leptin was analyzed using a microplate spectrophotometer (ThermoScientific, Chantilly, VA) at 540 nm.

### Implantation of osmotic minipump

Animals were anesthetized with pentobarbital sodium (50 mg/kg, IP) for implantation of osmotic minipump according to previously reported procedures [30, 34]. Briefly, a midline dorsal neck incision was made and the underneath muscle layers were dissected to expose the dura mater between the foramen magnum and C1 lamina. This was followed by perforation of dura with a 22-gauge steel needle and insertion of a PE-10 catheter (Clay Adams, Sparks, MD) into the cisterna magna. Patency of the implantation was assured by drainage of cerebrospinal fluid (CSF) from the outer end of catheter. The catheter was then sealed to the dura with tissue glue and the incision was closed with layered sutures. The outer end of the catheter was connected to a micro-osmotic minipump (Alzet 1007D; Durect Co., Cupertino, CA), which was placed under the skin in the neck region. Animals received procaine penicillin (1000 IU, IM) injection postoperatively, and only animals that showed progressive weight gain after the operation were used in subsequent experiments. Control infusion of artificial CSF (aCSF) served as the volume and vehicle control. The composition of aCSF was (mM): NaCl 117, NaHCO_3_ 25, Glucose 11, KCl 4.7, CaCl_2_ 2.5, MgCl_2_ 1.2 and NaH_2_PO_4_.

### RVLM tissue collection

At age of 10 or 12 weeks, ND and HFD offspring were deeply anesthetized with an overdose of pentobarbital sodium (100 mg/kg, IP), followed by intracardial infusion with warm normal saline. The skull was opened and brain stem was rapidly removed and immediately frozen on ice. The medulla oblongata covering rostral ventrolateral medulla (RVLM) was blocked between 0.5 and 1.5 mm rostral to the obex, based on the atlas of Watson and Paxinos [35], using a rodent brain matrix (World Precision Instruments, Sarasota, FL). Both sides of the ventrolateral medulla covering RVLM (approximately 1.5- to 2.5-mm lateral to the midline and medial to the spinal trigeminal tract) were collected by micropunches with a 1-mm inner diameter stainless-steel burr [30, 31, 34]. Medullary tissues collected were stored at − 80 °C for subsequent protein analysis.

### Total protein preparation

RVLM tissue samples were homogenized in ice-cold lysis buffer by a Dounce grinder with a tight pestle [30, 31, 34]. A cocktail of protease inhibitors (Sigma-Aldrich, St. Louis, USA) was included in the isolation buffer to prevent protein degradation. Solubilized proteins were centrifuged at 20,000×*g* at 4 °C for 15 min, supernatant was collected and total protein was quantified by the Bradford assay with a protein assay kit (Bio-Rad, Hercules, CA).

### Western blot analysis

Total protein extracted from RVLM samples was used to determine the expression levels of AT_1_R, gp91^phox^, p67^phox^ or p47^phox^ subunits of NADPH oxidase, isoforms of superoxide dismutase (SOD), glutathione peroxidase (GPx), catalase, nitric oxide synthase (NOS) isoforms, phosphorylated and total AMPK (p-AMPK and t-AMPK), sirtuin 1 (SIRT1), SIRT3, peroxisome proliferator-activated receptor gamma co-activator α (PGC-1α), mitochondrial transcription factor A (TFAM) or leptin by Western blot analysis. Protein samples were subject to 8–12% SDS-polyacrylamide gel electrophoresis and then transferred onto polyvinylidene difluoride transfer membranes (Immobilon-P membrane; Millipore, Bedford, MA) for 1.5 h at 4 °C, using a Bio-Rad miniprotein-III wet transfer unit (Bio-Rad). The transfer membranes were then incubated with a blocking solution (5% nonfat dried milk dissolved in Tris-buffered saline-Tween buffer (pH 7.6, 10 mM Tris-HCl, 150 mM NaCl, and 0.1% Tween 20) for 1 h at room temperature [34, 36].

The primary antisera used included goat polyclonal, rabbit polyclonal or monoclonal, or mouse monoclonal antiserum against AT_1_R (1:2000; Santa Cruz Biotechnology, Santa Cruz, CA), gp91^phox^ (1:5000; BD Biosciences, Sparks, MD), p67^phox^ or p47^phox^ (1:5000; Santa Cruz Biotechnology), manganese SOD (SOD1, 1:6000; Stressgen, Ann Arbor, MI), copper/zinc SOD (SOD2, 1:3000; Stressgen), extracellular SOD (SOD3, 1:5000; Stressgen), catalase (1:4000; Stressgen), GPx1 or GPx 3 (1:5000; BD Biosciences), NOS1, NOS2 or NOS3 (1:2000; BD Biosciences), t-AMPKα or p-AMPKα at Thr^172^ (1:1000; Cell Signaling, Danvers, MA), SIRT1 or SIRT3 (1:1000; Proteintech, Rosemont, IL), PGC-1α (1:1000; Santa Cruz Biotechnology), TFAM (1:1000; Abcam, Rockville, MA), leptin (1:1000; Abcam) or GAPDH (1:10,000; Merck). Membranes were washed three times with TBS-t buffer, followed by the secondary antibodies (1:10000; Jackson ImmunoResearch, West Grove, PA) for 1 h. This was followed by incubation with horseradish peroxidase-conjugated goat anti-rabbit IgG or goat anti-mouse IgG (Jackson ImmunoReserach). Specific antibody-antigen complex was detected using an enhanced chemiluminescence Western blot detection system (GE Healthcare Bio-Sciences Corp., Piscataway, NJ). The amount of detected proteins was quantified by ImageJ software (NIH, Bethesda, MD), and was expressed as the ratio to loading control (GAPDH).

### Measurement of ROS in RVLM

To measure the reactive oxygen species (ROS) in RVLM tissues, total protein extracted was reacted with the oxidation-sensitive fluorescent probe dihydroethidium (DHE, 1 μM; Invitrogen) [36]. RVLM tissues were homogenized in 20 mM sodium phosphate buffer (pH 7.4; containing 0.01 mM EDTA) by a glass-to-glass homogenizer. The homogenate was subjected to low speed centrifugation at 1000×*g* for 10 min at 4 °C. The supernatant was collected immediately for ROS measurement by reacting the lysate with DHE at 37 °C for 15 min under protection from light. The fluorescence signal was measured in a microplate reader (FluorStar; Biodirect, Inc., Taunton, MA). Specificity for superoxide was determined by the addition of a membrane-permeable superoxide dismutase SOD (350 U/mL) into the incubation medium. Protein sample extracted from RVLM of individual ND or HFD offspring was analyzed in triplicates and the results are shown as the mean of the three values.

### Measurement of lipid peroxidation in RVLM

Levels of lipid peroxidation were measured by a malondialdehyde (MDA) assay kit (Biovision, Milpitas, CA), following the protocol provided by the manufacturer. In brief, RVLM tissue was homogenized with MDA lysis buffer on ice to remove precipitated protein. The supernatant was then reacted with thiobarbituric acid. The level of cellular MDA was determined using a microplate spectrophotometer (ThermoScientific), with colorimetric absorbance read at 532 nm. Protein sample extracted from RVLM of individual ND or HFD offspring was analyzed in triplicates and the results are shown as the mean of the three values.

### Measurement of tissue NOx level

RVLM tissues were homogenized in lysis buffer. After centrifugation in 20,000×*g* at 4 °C for 15 min, the supernatant was deproteinized using a Centricon-30 filtrator (Microcon YM-30, Bedford, MA) and stored at − 80 °C until further processing. The level of total nitrate and nitrite (NOx) was determined with the purge system of a NO analyzer (Sievers NOA 280™, Boulder, CO) based on chemiluminescence reaction [36]. All assays were performed in triplicate and the results are shown as the mean of the three values.

### Determination of mitochondrial DNA copy number

Total RNA was extracted from RVLM tissue using TRIzol (Invitrogen, Carlsbad, CA) and cDNA was reversely transcribed from RNA (200 ng) using cDNA synthesis kit (EP0442, ThermoScientific). Mitochondrial DNA copy number was determined by the ratio of cDNA amplified from mtDNA-encoded NADH dehydrogenase subunit 1 (ND1) to nucleus-encoded 18S RNA genes. Primers for the ND1 probe correspond to nucleotides 389–408 (forward) and 572–592 (reverse; PCR product of 200 base pairs) of the rat mitochondrial genome (Chromosome MT - NC_001665.2). Primers for the 18S probe correspond to nucleotides 681–702 (forward) and 864–884 (reverse; PCR product of 200 base pairs) of the rat nuclear genome (Chromosome 14 - NC_005113.3). The primer sequences are: ND1, Forward (5′-3′) TCGGAGCCCTACGAGCCGTT /Reverse (5′-3′) AGGGAGCTCGATTTGTTTCTG; 18 s rRNA, Forward (5′-3′) TAGTTGGATCTTGGGAGCGGG /Reverse (5′-3′) CCGCGGTCCTATTCCATTATT.

Quantitative real-time polymerase chain reaction (qPCR) was performed by a Roche LightCycler 480 (Roche Applied Science, Mannheim, Germany) apparatus with LightCycler 480 SYBR Green I Master kit (Roche Applied Science). DNA sample (10 ng) from RVLM was mixed with 10 μL LightCycler 480 SYBR Green I Master Mix that contained forward and reverse primers (at a final concentration of 0.4 μM) in a final volume of 20 μl. The qPCR reactions were conducted as follows: initiation at 50 °C for 2 min, 95 °C for 1 min, 40 cycles of denaturation at 95 °C for 15 s, annealing at 60 °C for 20 s, extension at 72 °C for 15 s, and finally holding at 4 °C. The value of the threshold cycle number (Ct) of the 18 s rRNA gene and the ND1 gene was determined for each individual qPCR reaction. ΔCt = [Ct (ND1) - Ct (18S)] represents the relative abundance. The results are expressed as the copy number of mtDNA/sample by 2^-ΔCt^ [22]. Each measurement was performed in triplicate and the results are shown as the mean of the three values.

### Measurement of tissue ATP level

RVLM tissues were homogenized in a protein extraction solution (Pierce, Rockford, IL), followed by centrifugation at 10,000×*g* for 10 min. The supernatant was subject to determination of ATP concentration by an ATP colorimetric assay kit (Biovisoin) [22]. The ATP level was detected using a microplate reader (ThermoScientific) and was normalized to protein concentration of the sample. Each measurement was performed in triplicate and the results are shown as the mean of the three values.

### Double immunofluorescence staining

At age of 12 weeks, ND and HFD offspring were perfused transcardially with warm saline under deep sodium pentobarbital anesthesia (100 mg/kg, IP), and the brain stem was removed and post-fixed overnight in 4% paraformaldehyde, followed by 30% sucrose solution for at least 5 days. 30-μm coronal sections of the rostral medulla oblongata were cut using a cryostat (Leica, Nussloch, Germany). The sections were rinsed for 30 min in PBS. After pre-absorption in gelatin (0.375%), normal horse serum (3%) and triton-X 100 (0.2%) in PBS, the sections were incubated with a rabbit polyclonal antibody against AT_1_R (1:250; Santa Cruz Biotechnology), mouse monoclonal antibody against gp91^phox^ (1:250; BD Biosciences), rabbit polyclonal antibody against SOD2 (1:250; Biovision) or mouse monoclonal or rabbit polyclonal antibody against NeuN (1:2000; Millipore) at 4 °C overnight and then rinsed 3 times in PBS. After incubation in Alex 594 conjugated anti-mouse or rabbit IgG (1:1000; Molecular Probes), or Alex 488 conjugated anti-rabbit or mouse IgG (1:1000; Molecular Probes, Eugene, OR) for 1 h, the sections were rinsed 3 times in MilliQ distilled water. Sections were mounted and observed under a confocol microscope (FluoView FV10i; Olympus, Tokyo, Japan) [34, 37].

### Experimental design

One male rat was housed with two females until mating was confirmed by the observation of vaginal plug. Pregnant female rats were randomly assigned to receive normal diet (ND, 46% complex carbohydrate, 3.4 Kcal/g; Harlan Laboratories, Madison, WI) or HFD (60% fructose, 3.6 Kcal/g; TD.89247; Harlan Laboratories) chow during the entire period of pregnancy and lactation. Both food and water were provided ad libitum. Clinical observations indicate that men are more prone to hypertension at a younger age [38]. Accordingly, only male offspring from litters culled to sizes of eight pups after birth were used in subsequent experiments.

After weaning (3 weeks after birth), both ND and HFD offspring returned to ND chow, and body weight as well as food intake of offspring were measured and recorded once per week until age of 12 weeks. Metabolic indices were measured at 6, 9 and 12 weeks of age and BP was monitored weekly from age of 6 to 12 weeks (in some offspring to 18 weeks). At age of 8 weeks, ND and HFD offspring were randomly separated into groups to receive either oral administration via gavage of a 3-hydroxy-3-methylglutaryl coenzyme A (HMG-CoA) reductase inhibitor, simvastatin (5 mg·kg^− 1^·day^− 1^) or a AMPK activator, metformin (400 mg·kg^− 1^·day^− 1^); or microinfusion into the cistern magna of an AT_1_R antagonist, losartan (3 μg·μL^− 1^·h^− 1^). All treatments were applied for 4 weeks. Dosage of the compounds was adopted or modified from previous studies [28, 39] or determined in pilot experiments. During outcome assessments, treatment groups and assignments were masked from the experimenters.

### Drugs used

Drugs used include a HMG-CoA reductase inhibitor, simvastatin (5 mg·kg^− 1^·day^− 1^; Sigma-Aldrich), an AMPK activator, metformin (400 mg·kg^− 1^·day^− 1^; Sigma-Aldrich) or an AT_1_R antagonist, losartan (3 μg·μL^− 1^·h^− 1^; Sigma-Aldrich). Simvastatin and metformin were administered to the ND or HFD offspring via gastric gavage using a blunt-ended needle, whereas losartan was microinfused into the cistern magna via the implanted osmotic minipump. Normal saline served as volume and vehicle control for simvastatin and metformin, and artificial CFS for losartan.

### Statistical analysis

Data are expressed as means ± SEM. All statistical analyses were performed by the Graphpad Prism analysis software (La Jolla, CA). One-way or two-way ANOVA with repeated measures was used, as appropriate, to assess group means; to be followed by Tukey’s or Newman-Keuls multiple-range test for post hoc assessment of individual means. *P* value < 0.05 was considered statistically significant.

## Results

### Young offspring to maternal HFD exposure exhibit elevated blood pressure, augmented sympathetic vasomotor tone and higher circulatory norepinephrine level

**Fig. 1:**
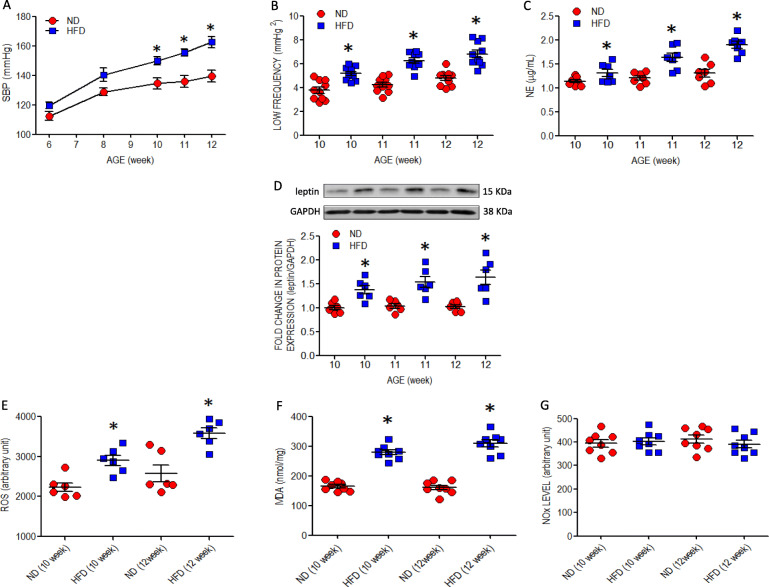
Cardio-metabolic parameters and redox signals at RVLM in young offspring exposed to maternal HFD or ND**.** Dams were exposed to a 60% HFD or ND during gestation and lactation. Both HFD and ND offspring returned to ND immediate after weaning (at age of 3 weeks). **a** SBP measured using tail-cuff plethysmography under conscious condition; **b** sympathetic vasomotor activity represented by power density of low frequency component of SBP signals; **c** plasma NE determined by high performance liquid chromatography; **d** protein expression of leptin in RVLM assessed by Western blot; **e** tissue level of ROS measured by dihydroethidium signal; **f** MDA determined by colorimetric spectrophotometry; and **g** NOx evaluated based on chemiluminescence reaction in RVLM. Analysis was performed on tissues collected bilaterally from individual RVLM. All measurements were made in ND (*n* = 6–12) and HFD (*n* = 6–12) offspring at age of 6, 8, 10, 11 and/or 12 weeks. Data are presented as mean ± SEM. *P < 0.05 versus ND group at comparable age in post hoc Tukey’s multiple range analysis. There is no significant difference between data shown in **G** in one-way ANOVA

In comparison with ND group, baseline SBP (Fig. 1a), sympathetic vasomotor activity, as reflected by the power density of the LF component of SBP signals (Fig. 1b), and circulatory NE level (Fig. 1c) were significantly increased in male, young offspring exposed to maternal HFD that became significant at age of 10 weeks. The elevated SBP (HFD vs. ND: 165.4 ± 5.7 vs. 141.9 ± 3.9 mmHg, *P* < 0.05, *n* = 8) was maintained in adult HFD offspring at age of 18 weeks. Maternal HFD also programmed the development of metabolic syndrome, characterized by increases in fasting plasma insulin, glucose, triglyceride and leptin levels, as well as HOMA index (Table S1). In addition, expression of leptin in RVLM (Fig. 1d) was also augmented.

### Maternal HFD leads to oxidative stress in RVLM of young offspring

**Fig. 2:**
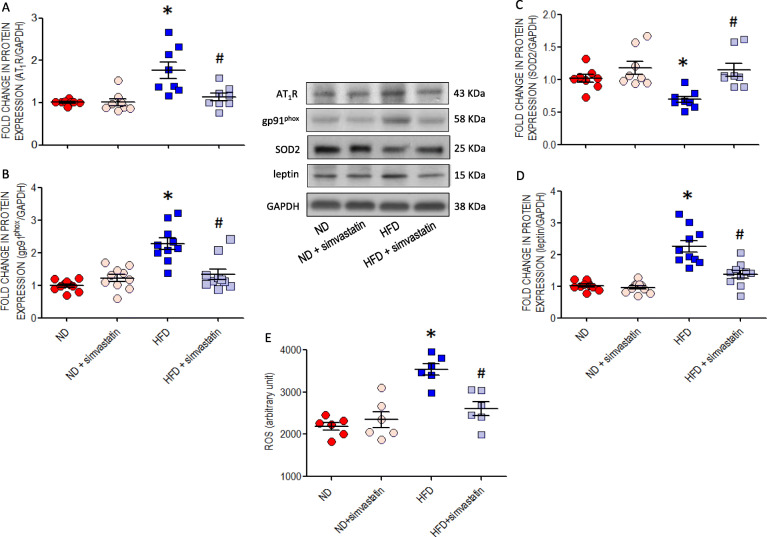
Expressions of AT_1_R, molecules involved in redox homeostasis and leptin in RVLM of young offspring exposed to maternal HFD or ND. Representative gels and densitometric analysis of results from Western blot showing changes in protein expression of (**a**) AT_1_R, (**b**) gp91^phox^ (**c**) SOD2, and (**d**) leptin or (**e**) ROS level in RVLM of ND (*n* = 6–10) or HFD (*n* = 6–10) offspring, alone or with additional treatment with a HMG-CoA reductase inhibitor, simvasatatin (5 mg·kg^− 1^·day^− 1^), administered via gastric gavage at age of 8 weeks for 4 weeks. Analysis was performed on tissues collected bilaterally from individual RVLM at age of 12 weeks. Data on protein expression were normalized to the average ND control value, which is set to 1.0, and are presented as mean ± SEM. *P < 0.05 versus ND group, ^#^*P* < 0.05 versus HFD group in post hoc Newman-Keuls multiple-range test

In tissues collected from RVLM at 10 or 12 weeks of age, offspring to HFD dams showed greater levels of ROS (Fig. 1e) and MDA (Fig. 1f), but not NO (Fig. 1g). These changes were associated with increases in protein expression of AT_1_R (Fig. 2a) or gp91^phox^ (Fig. 2b) and a decrease of SOD2 (Fig. 2c) in RVLM tissues and neurons (Figure S1) examined at 10 (data not shown) or 12 weeks. All these changes were alleviated by oral administration of simvastatin (5 mg·kg^− 1^·day^− 1^) to the offspring commencing at 8 weeks of age. The same treatment also mitigated ROS accumulation (Fig. 2e) and the increased leptin expression (Fig. 2d) in RVLM, as well as the increases in fasting plasma triglyceride and leptin (Table S2) at age of 12 weeks. On the other hand, no apparent change in the expression of p47^phox^, p67^phox^, SOD1, SOD3, catalase, GPx, or NOS isoforms (Figure S2) was detected in the RVLM of HFD offspring at comparable ages.

### Maternal HFD impairs mitochondrial biogenesis in RVLM of young offspring

**Fig. 3:**
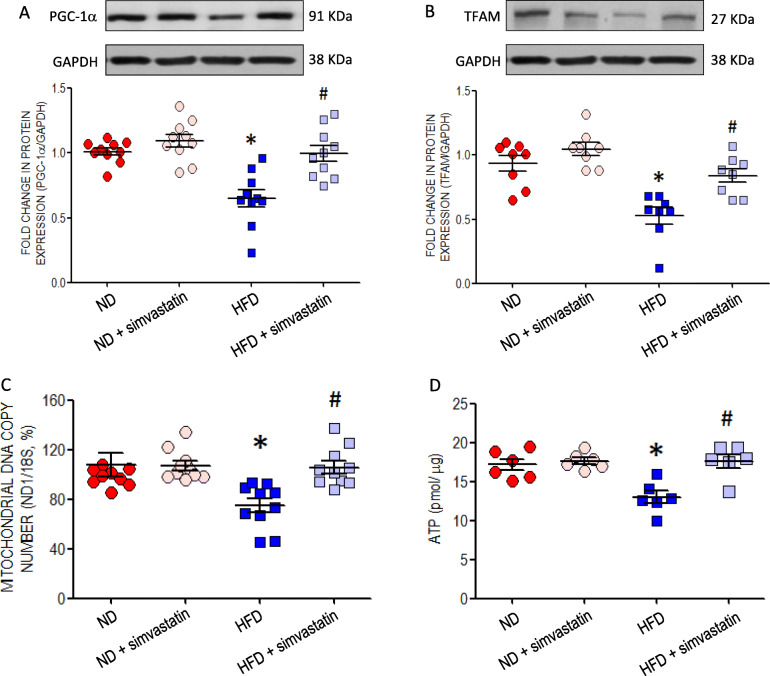
Mitochondrial biogenesis in RVLM of young offspring exposed to maternal HFD or ND. Representative gels (insets) and densitometric analysis of results from Western blot showing changes in protein expression of (**a**) PGC-1α (*n* = 10 per group) and (**b**) TFAM (*n* = 8 per group), two key molecules in transcription and replication of mitochondrial DNA; (**c**) mitochondrial DNA copy number (*n* = 10 per group); and (**d**) tissue content of ATP (*n* = 6 per group) in RVLM of ND or HFD offspring, alone or with additional treatment with simvastatin (5 mg·kg^− 1^·day^− 1^), administered via gastric gavage at age of 8 weeks for 4 weeks. Analysis was performed on tissues collected bilaterally from individual RVLM at age of 12 weeks. Data on protein expression were normalized to the average ND control value, which is set to 1.0, are presented as mean ± SEM. *P < 0.05 versus ND group ^#^*P* < 0.05 versus HFD group in post hoc Newman-Keuls multiple-range test

Compared to ND offspring, the expression of PGC-1α (Fig. 3a) and TFAM (Fig. 3b), two key molecules in transcription and replication of mitochondrial DNA [40], together with mitochondrial DNA copy number (Fig. 3c) were decreased in RVLM of HFD offspring at 10- (data not shown) and 12-week old. These changes are accompanied by a significant decrease in tissue content of ATP (Fig. 3d). Oral administration of simvastatin (5 mg·kg^− 1^·day^− 1^) at age of 8 weeks for 4 weeks significantly restored the protein expression of PGC-1α and TFAM, and maintained tissue ATP content in RVLM of HFD offspring at 12 weeks of age.

### Maternal HFD suppresses AMPK activation and SIRT expression in RVLM of young offspring

**Fig. 4:**
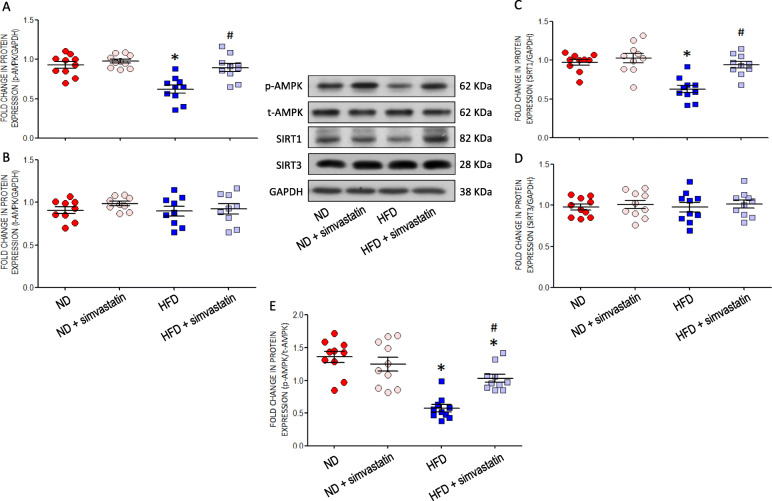
AMPK/SIRT signals in RVLM of young offspring exposed to maternal HFD or ND. Representative gels and densitometric analysis of results from Western blot showing changes in protein expression of (**a**) p-AMPK, (**b**) t-AMPK, (**c**) SIRT1 and (**d**) SIRT3, as well as (**e**) ratio between p-AMPK/t-AMPK in RVLM of ND or HFD offspring, alone or with additional treatment with simvasatatin (5 mg·kg^− 1^·day^− 1^), administered via gastric gavage at age of 8 weeks for 4 weeks. Analysis was performed on tissues collected bilaterally from individual RVLM at age of 12 weeks. Data on protein expression were normalized to the average ND control value, which is set to 1.0, and are presented as mean ± SEM (*n* = 10 in all groups). *P < 0.05 versus ND group ^#^*P* < 0.05 versus HFD group in post hoc Newman-Keuls multiple-range test

RVLM tissue collected from HFD offspring at 10 (data not shown) or 12 weeks of age showed reduced expression of phosphorylated AMPK at the Thr^172^ residue (p-AMPK) (Fig. 4a) and SIRT1 (Fig. 4c), but not total AMPK (t-AMPK) (Fig. 4b) or SIRT3 (Fig. 4d), along with a decreased p-AMPK/t-AMPK ratio (Fig. 4e); all of which were partially protected by oral administration of simvastatin (5 mg·kg^− 1^·day^− 1^) to the offspring at 8 weeks of age.

### Activation of AMPK retards AT_1_R upregulation and restores the impaired mitochondrial biogenesis in RVLM of young HFD offspring

**Fig. 5:**
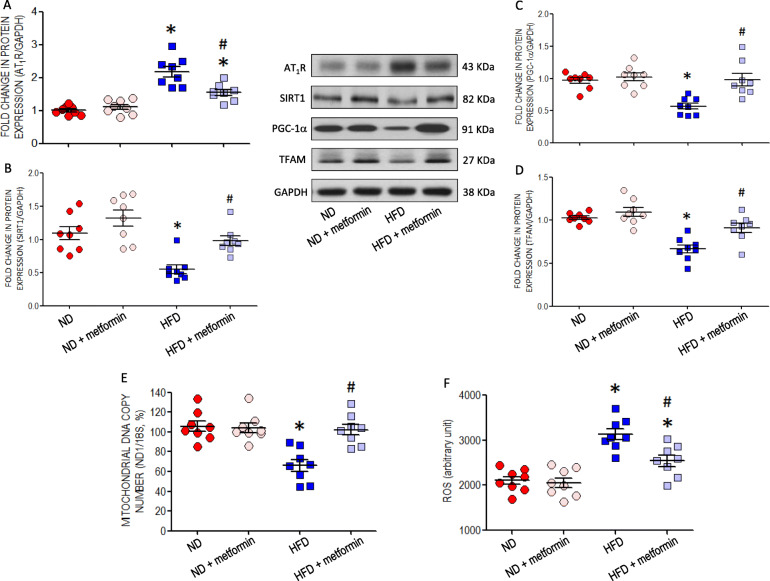
AMPK activation on the expressions of AT_1_R and SIRT1, mitochondrial biogenesis and ROS level in RVLM of young offspring exposed to maternal HFD or ND. Representative gels and densitometric analysis of results from Western blot showing changes in protein expression of (**a**) AT_1_R; (**b**) SIRT1, (**c**) PGC-1α, or (**d**) TFAM; (**e**) mitochondrial DNA copy number; and (**f**) ROS level in RVLM of ND or HFD offspring, alone or with additional treatment with an AMPK activator, metformin (400 mg·kg^− 1^·day^− 1^), administered via gastric gavage at age of 8 weeks for 4 weeks. Analysis was performed on tissues collected bilaterally from individual RVLM at age of 12 weeks. Data on protein expression were normalized to the average ND control value, which is set to 1.0, and are presented as mean ± SEM (*n* = 8 in all groups). *P < 0.05 versus ND group and ^#^*P* < 0.05 versus HFD group in post hoc Newman-Keuls multiple-range test

Oral administration of an AMPK activator, metformin (400 mg·kg^− 1^·day^− 1^), for 4 weeks at age of 8 weeks significantly retarded the increased AT_1_R (Fig. 5a), restored the decreased SIRT1 (Fig. 5b), PGC-1α (Fig. 5c) or TFAM (Fig. 5d) expression and maintained mitochondrial DNA copy number (Fig. 5e) in RVLM of offspring exposed to maternal HFD. The same treatment also significantly ameliorated the heightened ROS production in RVLM of HFD offspring (Fig. 5f).

### Protection against RVLM oxidative stress lessens increase in SBP in young HFD offspring

**Fig. 6:**
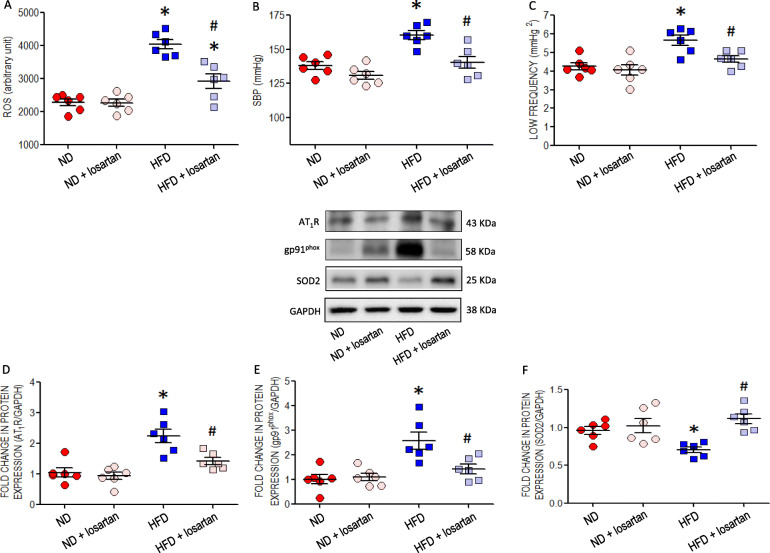
Effects of AT_1_R antagonist on blood pressure, sympathetic vasomotor activity, ROS level and expressions of AT_1_R, gp91phox or SOD2 protein in RVLM of young offspring exposed to maternal HFD or ND. **a** tissue ROS level; **b** SBP; **c** sympathetic vasomotor activity; and representative gels and densitometric analysis of results from Western blot showing changes in protein expression of (**d**) AT_1_R, (**e**) gp91^phox^, or (**f**) SOD2 in RVLM of ND or HFD offspring, alone or with additional treatment with an AT1R antagonist, losartan (3 μg·μL^− 1^·h^− 1^), microinfused via a minipump into the cistern magna at age of 8 weeks for 4 weeks. Analysis was performed on tissues collected bilaterally from individual RVLM at age of 12 weeks. Data on protein expression were normalized to the average ND control value, which is set to 1.0, and are presented as mean ± SEM (*n* = 6 in all groups). *P < 0.05 versus ND group and ^#^*P* < 0.05 versus HFD group in post hoc Newman-Keuls multiple-range test

Infusion into the cistern magna of an AT_1_R antagonist, losartan (3 μg·μL^− 1^·h^− 1^), to young offspring from age of 8 weeks for 4 weeks effectively diminished the augmented ROS production (Fig. 6a), as well as the increase in SBP (Fig. 6b) and power density of LF component of SBP signal (Fig. 6c) manifested after exposure to maternal HFD. The same treatment also reversed the increased AT_1_R (Fig. 6d) and gp91^phox^ (Fig. 6e), as well as the reduced SOD 2 (Fig. 6f) expression in RVLM of HFD offspring. Losartan, on the other hand, exerted no effect on protein expressions of p-AMPK, SIRT1 or p-AMPK/t-AMPK ratio, all of which were suppressed in the HFD offspring (Figure S3). Fasting plasma insulin, glucose, triglyceride or leptin (Table S2) levels measured at age of 12 weeks was not affected by intracisternal infusion of losartan.

### Protection against increase of SBP in young adult HFD offspring by simvastatin and metformin

**Fig. 7:**
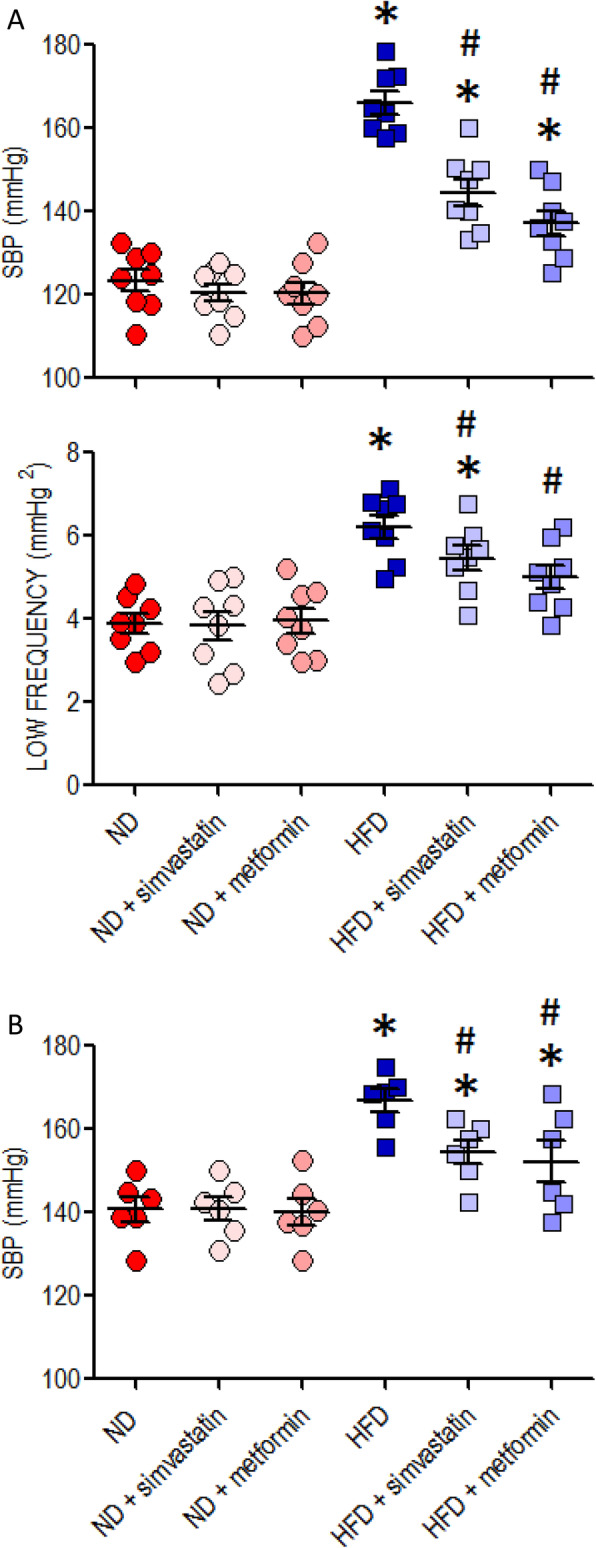
Reprogramming hypertension in young HFD offspring by simvastatin and metformin. Changes in (**a**) SBP and sympathetic vasomotor activity at age of 12 weeks or (**b**) SBP at age of 18 weeks in ND or HFD offspring following 4-week treatment with simvastatin (5 mg·kg^− 1^·day^− 1^) or metformin (400 mg·kg^− 1^·day^− 1^), administered via gastric gavage at age of 8 weeks for 4 weeks. Data are presented as mean ± SEM (*n* = 6–8 per group). *P < 0.05 versus ND group and ^#^*P* < 0.05 versus HFD group in post hoc Newman-Keuls multiple-range test

Oral administration of simvastatin (5 mg·kg^− 1^·day^− 1^) or metformin (400 mg·kg^− 1^·day^− 1^) from 8 weeks of age effectively reduced the increase in SBP and the heightened sympathetic vasomotor activity (Fig. 7a) in young HFD offspring at age of 12 weeks. The same treatments also lessened the programmed hypertension (Fig. 7b) measured at 18 weeks of age.

## Discussion

The most significant findings of the present study are: (1) maternal exposure to HFD during gestation and lactation increases tissue oxidative stress in RVLM, priming the increases in sympathetic vasomotor activity and SBP in young offspring; (2) the programmed tissue oxidative stress in RVLM of HFD offspring are attributed to the increase in AMPK-regulated AT_1_R expression and impairment of SIRT1-associated mitochondrial biogenesis; (3) the perturbed redox homeostasis in RVLM of young HFD offspring could be significantly restored by oral administration of simvastatin or metformin; (4) the same treatments reprogram the hemodynamic dysfunctions observed in young HFD offspring, and lessens the programmed hypertension in adult life. Together, these data offer new documentation in support of oxidative stress in RVLM in fetal programming of hypertension in adult offspring that are exposed to maternal HFD, and provide novel insights into the roles of AT_1_R and mitochondria biogenesis, via the dysregulation of AMPK/SIRT1 signaling, in this process.

Hypertension is a major risk factor for cardio-vascular disease, the leading cause of morbidity and mortality worldwide. Emerging evidence from both epidemiological and animal studies indicates that the pathological process of hypertension in adult life may have its origin at pre- and neonatal stages. This concept of fetal programming of hypertension was first postulated by Barker [3] based on the seminal observations of an inverse relationship between infant birth weight and SBP in adult humans. Numerous experimental studies that mimic intrauterine nutritional insults have thereafter been utilized to investigate the etiology of programmed hypertension. Among the multiple mechanisms proposed, activation of the SNS represents a key pathophysiological process [15, 16]. In line with this postulation, we found in the present study that the increase in SBP of young offspring exposed to maternal HFD was accompanied by a parallel increase in plasma NE level, suggesting an activation of peripheral SNS. The observed augmentation of sympathetic vasomotor activity further indicated the involvement of brain machinery in the sympathoexcitation of HFD offspring.

Both oxidative stress and nitrosative stress in RVLM, where the sympathetic premotor neurons reside, have been demonstrated to mediate sympathoexcitation and promote hypertension [18, 19, 23]. Based on a maternal HFD model of programmed hypertension, our results revealed that oxidative rather than nitrosative stress in RVLM may account for the increase in sympathetic vasomotor activity observed in young offspring. We found that the tissue level of ROS and its distribution in RVLM neurons were enhanced; whereas no apparent change in NO level or expression of NOS isoforms was detected in HFD offspring. At the molecular level, the elevated ROS level in RVLM was attributed by an increase in gp91^phox^ subunit of the NADPH oxidase and downregulation of the antioxidant SOD2, suppression of PGC-1α and TFAM expression, together with decrease in mitochondrial DNA copy number, events known to underpin sympathoexcitation in animal models of hypertension [18–23]. It is likely that activation of AT_1_R plays a pivotal role in priming tissue oxidative stress in RVLM of HFD offspring. Augmented expression of the receptor protein was induced by maternal HFD. More importantly, inhibition of AT_1_R action by losartan reversed the induced molecular changes and ameliorated ROS accumulation in RVLM of HFD offspring. Deficiencies in NO production, eNOS expression, NOS coupling and/or NO-cGMP signaling in the kidney [8, 14, 41] and vasculature [42] have been reported as potential underlying mechanisms for fetal programming of hypertension. Whether nitrosative stress in RVLM occurs in HFD offspring at an age earlier or later than that studied in the present study is not immediately clear and awaits further interrogation.

The brain renin-angiotensin system is an important element of hypertension programming during fetal life. Not only the AT_1_R expression in the brain areas involved in cardiovascular regulation is increased in fetus to antenatal nutrient deprivation, intracerebroventricular injection of AT_1_R antagonist significantly reduces the increase in mean arterial BP of the offspring [43], although the underlying regulatory mechanism is unknown. In addition to being a key regulator in whole-body energy metabolism [24, 25], AMPK, an evolutionarily conserved serine/threonine kinase, exhibits pluripotent cellular functions. In cultured cardiomyocytes, activation of AMPK reduces angiotensin II (Ang II)-induced AT_1_R upregulation and protects the cells from Ang II-induced hypertrophy [44]. AMPK is expressed in various tissues, including the brain, although there is currently no study that elucidates the relationship between AMPK and AT_1_R in neural tissues. Interestingly, we found in the present study an inverse relationship between AT_1_R expression and AMPK phosphorylation in RVLM of HFD offspring. That the induced increase in AT_1_R expression was significantly inhibited by treatment with an AMPK activator, metformin, further suggests that AT_1_R upregulation could be consequential to the blunted AMPK signals programmed by maternal HFD. This relationship between AMPK and AT1R is further supported by the observations in which AT_1_R blockade exerts negligible effect on the suppressed protein expressions of p-AMPK and SIRT1 in RVLM of HFD offspring. Compromised AMPK signals were recently demonstrated to be associated with intolerance to cardiac insult in offspring of gestational diabetes mellitus [45]. Phosphorylated AMPK was postulated to reduce AT_1_R levels through down-regulation of genes encoding the receptor via the PGC-1α/PPARγ pathway [46]. It is noteworthy that AMPK exhibits multiple phosphorylation sites on its catalytic subunit α, including the phosphorylation of AMPKα1 on Thr^172^ and AMPKα1/α2 on Ser^471^/Ser^491^ [47]. Depending on the residues and/or sites of phosphorylation, as well as its cellular location, AMPK may exert different cellular actions. In this regard, AMPKα2 gene knockout significantly exacerbates the degree of transverse aortic constriction-induced left ventricle hypertrophy and dysfunction, which are not affected by AMPKα1 gene knockout [48]. At the same time, the metformin-promoted cardioprotection against ischemia/reperfusion injury is mediated via activation of both cytosolic AMPKα1 and nuclear AMPKα2 [49]. Therefore, a full understanding in the roles of AMPK in RVLM on programmed hypertension in HFD offspring remains to be unraveled.

Upon activation at the Thr^172^ residue, AMPK increases NAD^+^/NADH ratio, which in turn stimulates the NAD^+^-dependent deacetylases sirtuins [50], a family of mammalian class III histone deacetylases with implicated functions in health span and longevity [51]. SIRT1 is the most studied sirtuins and may exert cellular protective actions by promoting mitochondrial biogenesis and triggering the turnover of damaged mitochondria [52]. The present study provides novel evidence to suggest that dysregulated SIRT1-dependent mitochondrial biogenesis may play an active role in oxidative stress programmed by maternal HFD exposure in RVLM. As the master regulator of mitochondrial biogenesis, PGC-1α drives transcription and replication of mitochondrial DNA via activation of transcription factors on the promoter region of TFAM [53]. SIRT1, on the other hand, is known to deacetylate PGC-1α to increase its transcriptional activity [54]. It thus is intriguing that we found the expression of SIRT1 protein was significantly decreased, alongside reduced PGC-1α and TFAM expressions and mitochondrial DNA copy number in RVLM of HFD offspring. The expression of SIRT3, another major mitochondrial sirtuin [55], on the other hand, was not affected by the same maternal exposure. We further found that activation of AMPK with metformin mitigated the impaired mitochondrial biogenesis, suggesting that the action of SIRT1 is downstream to AMPK in the signaling cascade. Accordingly, in the presence of metformin, AMPK-induced SIRT1 expression may prevent the reduced expression of PGC-1α and TFAM primed by maternal HFD exposure, resulting in the maintenance of mitochondrial biogenesis and redox homeostasis in RVLM of HFD offspring. These findings confer a novel role for the dysfunctional AMPK/SIRT1 signaling in fetal programming of oxidative stress in RVLM from maternal HFD insult. Dysregulated AMPK-PGC-1α signal has recently been reported to be engaged in adult hypertension programmed by prenatal NO deficiency plus postnatal high-fat diet exposure [56]. In addition to biogenesis impairment, mitochondrial ROS generated from curtail in bioenergetics [19, 21, 30, 31] and activation of cyclophilin D (a regulatory subunit of the mitochondrial permeability transition pore) [57] also contribute to hypertension development. Moreover, it is interesting to note that cyclophilin D interacts with heat shock protein 90 [58] Bcl-2, p53 [59] and PPARα [60] in mediating AMPK activation. As such, additional studies are required to further explore the relationship of AMPK activation, mitochondrial dysfunction and ROS production in developmental programming of hypertension.

An intriguing finding in the present study is that both the increased AT_1_R expression and impaired mitochondrial biogenesis in RVLM of HFD offspring were ameliorated after 4 weeks of oral administration of simvastatin, a widely used statin in the treatment of cardio-metabolic diseases because of its lipid-lowering effect. These observations suggest that ROS accumulation in RVLM could be primed by peripheral dyslipidemia developed in young offspring to maternal HFD. In this regard, we found both plasma and RVLM leptin levels were increased in young HFD offspring. Moreover, oral simvastatin treatment abolished the higher leptin expression in RVLM, pointing to a permissive role of dyslipidemia in brain uptake of leptin. Leptin has been shown to be transported across the blood-brain barrier to the brain in mice fed high fat diet [61], and the increase in brain leptin level inhibits AMPK phosphorylation [62]. In addition, leptin receptors are colocalized with AT_1A_R in brain tissue [63]. Central infusion of leptin increases AT_1_R mRNA expression in forebrain cardiovascular structures [64]. The mechanisms underpinning the effect of leptin on AMPK phosphorylation and AT_1_R expression in RVLM of HFD offspring, however, require further elucidation. In addition, despite that simvastatin treatment ameliorates the augmented leptin expression in RVLM and that high level leptin inhibits AMPK phosphorylation [62], there is no evidence from the current study for an active role of simvastatin-regulated AMPK-SIRT1 signal in RVLM on dyslipidemia and hyperleptinemia manifested in HFD offspring.

Another salient finding of the present study is the functional impact of the anomalous AMPK-regulated AT_1_R expression and SIRT1-dependent mitochondrial biogenesis on the programming of elevated BP in young HFD offspring. We found that metformin, at treatment regimen that restored the AMPK-regulated AT_1_R overexpression and SIRT1-dependent impairment of mitochondrial biogenesis in RVLM, could significantly abolish sympathoexcitation and increase of SBP in young HFD offspring. Similar responses were evoked by intracisternal infusion of losartan; further emphasizing the importance of AT_1_R of RVLM in fetal programming of hypertension in HFD offspring. The reprogramming of SBP in young HFD offspring after oral administration of simvastatin was interpreted to imply a sympathoexcitatory role of leptin in RVLM. In this regard, RVLM neurons express functional leptin receptors and can respond to leptin by increasing renal sympathetic activity and mean arterial BP [65].

There are a few cautionary notes in conjunction with the present study. First, while animal studies have identified the adverse effects of maternal fructose consumption on fetus and disease risk in offspring, only a small number of human studies have shown an association between excessive fructose consumption with poor pregnancy outcome [66]. As such, the translational implication of the present study remains to be realized. Second, the susceptibility to develop cardio-metabolic dysfunctions in offspring to maternal intrauterine insults could be sex dependent with a higher prevalence in male [67, 68], limiting the use of proposed reprogramming interventions to treat female offspring. Third, the beneficial effects of the studied compounds could be mediated via mechanisms not deciphered in the present study. For example, since AMPK is also a key regulator of PGC-1α [69], metformin may exert its beneficial effect on tissue oxidative stress directly via activation of PGC-1α. In addition, cardiovascular protective action of simvastatin is related to its pleiotropic effect such as inhibition of Rho kinase and increasing NO in the RVLM [70]. Finally, with the exception of losartan, all studied compounds were given via oral administration; therefore, site of action (central versus peripheral) and the proposed signaling cascades of their protective effects await further confirmation. To this end, simvastatin is known to penetrate the blood-brain barrier [71], and brain infusion of simvastatin protects against heart failure through normalization of sympathetic outflow from RVLM [70].

## Conclusions

**Fig. 8:**
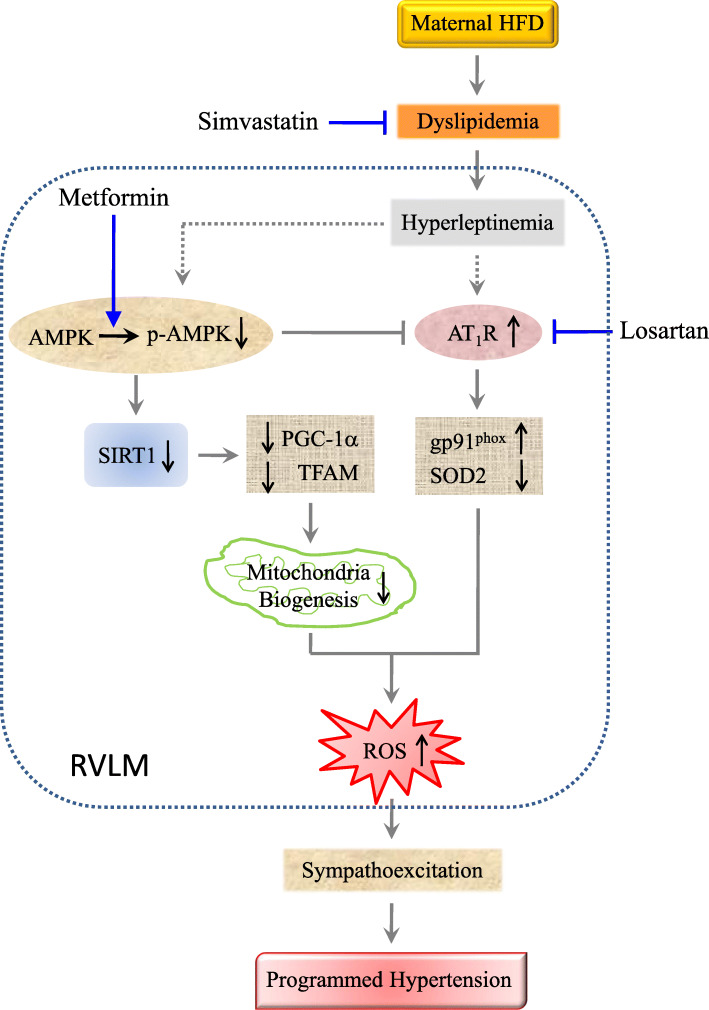
Schematic depiction of the contribution of AMPK/SIRT1 signaling in RVLM to oxidative stress-associated programming of hypertension in young offspring exposed to maternal high fructose. Circulatory dyslipidemia programmed by maternal HFD exposure during gestation and lactation increases leptin level, which may in turn increase AT_1_R expression and inhibit AMPK phosphorylation in RVLM. AT_1_R overexpression leads to tissue oxidative stress via an increase in gp91^phox^ and decrease in SOD2 expression. Suppressed AMPK activation results in inhibition of SIRT1 expression and its downstream signals, PGC-1α and TFAM, followed by tissue oxidative stress through the reduction in mitochondrial biogenesis. Suppressed AMPK activation also augments AT_1_R expression that promotes further oxidative stress. Accumulated ROS in RVLM contributes to the programmed hypertension in young HFD offspring via sympathoexcitation. The hypertension programming in HFD offspring could be protected by treatments with simvastatin to reduce dyslipidemia and hyperleptinemia; metformin to activate the AMPK/SIRT1 signals, and losartan to antagonize AT_1_R activation in RVLM. Arrows indicate activation; bar-headed lines indicate inhibition. Solid line denotes data from the present study or existing literature; dotted line denotes potential connections that require further documentation

In conclusion, the present study provides novel evidence to suggest that anomalous AMPK-regulated AT_1_R expression and SIRT1-mediated mitochondrial biogenesis may contribute to tissue oxidative stress in RVLM, which in turn primes the increases in sympathetic vasomotor activity and BP in young offspring exposed to maternal HFD exposure (Fig. 8). Furthermore, the impaired nutrient sensing signaling in RVLM may be initiated by hyperleptinemia in young offspring to maternal HFD exposure.

## Perspective

The burden of hypertension is an increasingly pressing matter because of the rising prevalence of higher BP in adolescence and its tracking into adult hypertension. Determining its pathogenesis to gain insights into its current and emerging management, particularly at young age, is therefore crucial and timely. Herein, in support of the DOHaD concept, our study highlights the transgenerational detrimental influence of maternal HFD on the pathogenesis of hypertension in young offspring. Our results suggest that programmed hypertension could be developed via common (e.g., activation of the SNS) or specific (e.g., dysfunction in AMPK/SIRT signaling) underlying mechanisms. Therapeutic remedies against programmed hypertension and cardio-metabolic disease should therefore be based on the underlying disease etiology rather than the disease phenotypes. Our observations that treatment with simvastatin or metformin to young offspring (at age of 8 weeks) protects against hypertension manifested in adult life (at age of 18 weeks) further emphasize a window of opportunity to treat adult cardio-metabolic disease of developmental origin at young age. Future studies examining the long-term beneficial effect on adult cardiovascular diseases of prenatal or perinatal treatment are crucial. It is also important to recommend dietary guidelines on fructose intake during pregnancy and lactation.

## Supplementary information

**Additional file 1: Table S1.** Metabolic indices of offspring to maternal ND or HFD exposure. **Table S2.** Effect of simvastatin or losartan on metabolic indices of young ND or HFD offspring.

**Additional file 2: Figure S1.** Representative laser-scanning confocal microscopic images showing the distribution of (**A**) AT_1_R, (**B**) gp91p^phox^ or (**C**) SOD2 (green fluorescence) in cells that were stained positively for a neuronal marker, neuron-specific nuclear protein (NeuN) (red fluorescence) in RVLM of ND or HFD offspring at age of 12 weeks. Scale bar: 50 μm. **Figure S2.** Representative gels (insets) and densitometric analysis of results from Western blot analysis showing changes in protein expression of (**A**) p47^phox^ or p67^phox^ of the NADPH oxidase, (**B**) SOD1 or SOD3, (**C**) catalase or GPx, or (**D**) NOS1–3 in RVLM of ND (*n* = 6–10) or HFD (*n* = 6–10) offspring, alone or with additional treatment with a HMG-CoA inhibitor, simvastatin (5 mg·kg^− 1^·day^− 1^), administered via gastric gavage at age of 8 weeks for 4 weeks. Analysis was performed on tissues collected bilaterally from individual RVLM at age of 12 weeks. Data on protein expression were normalized to the average ND control value, which is set to 1.0, and are presented as mean ± SEM. No significance difference among all groups in one-way ANOVA. **Figure S3.** Representative gels (insets) and densitometric analysis of results from Western blot analysis showing changes in protein expression of (**A**) p-AMPK and (**B**) SIRT1, as well as (**C**) ratio between p-AMPK/t-AMPK in RVLM of ND or HFD offspring, alone or with additional treatment with an AT1R antagonist, losartan (3 μg·μL^− 1^·h^− 1^), microinfused via a minipump into the cistern magna at age of 8 weeks for 4 weeks. Analysis was performed on tissues collected bilaterally from individual RVLM at age of 12 weeks. Data on protein expression were normalized to the average ND control value, which is set to 1.0, and are presented as mean ± SEM (*n* = 10 in all groups). *P < 0.05 versus ND group in post hoc Newman-Keuls multiple-range test. Group data of p-AMPK and SIRT1 in ND and HFD offspring from Fig. 4 are adopted for statistical comparison.

## Data Availability

Analytic methods, and study materials will be made available on publication of this research article. The data will be available from the corresponding author on reasonable request.

## Abbreviations

AMPK: AMP-activated protein kinase
AT_1_R: Angiotensin type 1 receptor
BP: Blood pressure
CSF: Cerebrospinal fluid
DHE: Dihydroethidium
DOHaD: Developmental original of adult health and disease
GPx: Glutathione peroxidase
HFD: High fructose diet
HMG-CoA: 3-hydroxy-3-methylglutaryl coenzyme A
HOMA: Homeostasis model assessment
IR: Insulin resistance
LF: Low frequency
MDA: Malondialdehyde
NADPH oxidase: Nicotinamide adenine dinucleotide diphosphate oxidase
ND: Normal diet
ND1: NADH dehydrogenase subunit 1
NE: Norepinephrine
NO: Nitric oxide
NOS: NO synthase
NOx: Nitrate and nitrite
OPA: O-phthaldehyde
PGC-1α: Peroxisome proliferator-activated receptor gamma co-activator α
qPCR: Quantitative real-time polymerase chain reaction
ROS: Reactive oxygen species
RVLM: Rostral ventrolateral medulla
SAP: Systemic arterial pressure
SBP: Systolic blood pressure
SD: Sprague-Dawley
SIRT: Sirtuin
SNS: Sympathetic nervous system
SOD: Superoxide dismutase
TFAM: Mitochondrial transcription factor A
