# Supplementary material for: Anomalous AMPK-regulated angiotensin AT1R expression and SIRT1-mediated mitochondrial biogenesis at RVLM in hypertension programming of offspring to maternal high fructose exposure
Source: J Biomed Sci. 2020 May 23;27:68. doi: 10.1186/s12929-020-00660-z (PMC7245869; doi:10.1186/s12929-020-00660-z)
Supplement: Supplementary file 1 — Additional file 1: Table S1. Metabolic indices of offspring to maternal ND or HFD exposure. Table S2. Effect of simvastatin or losartan on metabolic indices of young ND or HFD offspring. [file 12929_2020_660_MOESM1_ESM.docx]

# Table S1. Metabolic indices of offspring to maternal ND or HFD exposure.

| Maternal diet |  | ND |  |  | HFD |  |
| --- | --- | --- | --- | --- | --- | --- |
| Age (week) | 6 | 9 | 12 | 6 | 9 | 12 |
| Body weight (g) | 164.7±13.8 | 302.8±19.0 | 377.9±17.2 | 168.5±12.1 | 314.7±11.6 | 393.4±18.2^*^ |
| Triglyceride (mmol·L^-1^) | 1.14±0.09 | 1.12±0.12 | 1.09±0.10 | 1.13±0.15 | 1.24±0.17 | 1.30±0.16^*^ |
| Glucose (mg·dL^-1^) | 85.45±2.68 | 91.64±2.47 | 89.95±4.14 | 84.64±3.24 | 91.55±4.08 | 93.59±3.36^*^ |
| Insulin (pmol·L^-1^) | 34.97±1.91 | 43.41±1.61 | 45.88±4.02 | 34.82±1.53 | 46.98±3.48^*^ | 52.63±4.76^*^ |
| HOMA-IR | 0.63±0.07 | 0.65±0.10 | 0.88±0.08 | 0.62±0.06 | 0.79±0.11 | 0.95±0.15^*^ |
| Leptin (ng·mL^-1^) | 0.51±0.07 | 1.15±0.19 | 1.42±0.08 | 0.49±0.19 | 1.20±0.21 | 1.84±0.27^*^ |

| All measurements were taken at age of 6, 9, and 12 weeks from offspring exposed to maternal normal diet (ND; n = 12) or high fructose diet (HFD; n = 12). Data are presented as mean ± SEM. **P*<0.05 versus ND group at comparable age in the Student’s *t*-test. HOMA-IR, homeostasis model assessment for insulin resistance. |
| --- |
|  |
|  |
|  |

# Table S2. Effect of simvastatin or losartan on metabolic indices of young ND or HFD offspring.

| Maternal diet |  | ND |  |  | HFD |  |
| --- | --- | --- | --- | --- | --- | --- |
| Offspring treatment |  | simvastatin | losartan |  | simvastatin | losartan |
| Age (week) | 12 | 12 | 12 | 12 | 12 | 12 |
| Body weight (g) | 377.9±17.2 | 379.3±14.6 | 381.2±15.6 | 393.4±18.2^*^ | 389.4±13.4^*^ | 390.7±17.8^*^ |
| Triglyceride (mmol·L^-1^) | 1.09±0.10 | 1.13±0.11 | 1.05±0.14 | 1.30±0.16^*^ | 1.10±0.13^#^ | 1.27±0.15^*^ |
| Glucose (mg·dL^-1^) | 89.95±4.14 | 86.80±3.71 | 88.79±4.21 | 93.59±3.36^*^ | 92.85±3.62^*^ | 94.02±4.10^*^ |
| Insulin (pmol·L^-1^) | 45.88±4.02 | 47.85±2.43 | 46.75±3.73 | 52.63±4.76^*^ | 49.72±3.04^*^ | 51.92±5.01^*^ |
| HOMA-IR | 0.88±0.08 | 0.90±0.09 | 0.89±0.07 | 0.95±0.15^*^ | 0.93±0.12^*^ | 0.94±0.09^*^ |
| Leptin (ng·mL^-1^) | 1.42±0.08 | 1.33±0.11 | 1.46±0.09 | 1.84±0.27^*^ | 1.50±0.19^#^ | 1.88±0.31^*^ |

# Simvastatin (5 mg⋅kg^-1^⋅day^-1^, n = 10 per group) was administered via gastric gavage and losartan (3 μg⋅μL^-1^⋅h^-1^, n = 6 per group) was microinfused into the cisterna magna, commencing at age of 8 weeks for 4 weeks. Data are presented as mean ± SEM. **P*<0.05 versus ND group, ^#^P<0.05 versus HFD group at comparable age in the Student’s *t*-test. Values of ND and HFD offspring at age of 12 weeks are adopted from Table S1 for comparison. HOMA-IR, homeostasis model assessment for insulin resistance.
