# Supplementary material for: Anomalous AMPK-regulated angiotensin AT1R expression and SIRT1-mediated mitochondrial biogenesis at RVLM in hypertension programming of offspring to maternal high fructose exposure
Source: J Biomed Sci. 2020 May 23;27:68. doi: 10.1186/s12929-020-00660-z (PMC7245869; doi:10.1186/s12929-020-00660-z)

**Figure S1.** Representative laser-scanning confocal microscopic images showing the distribution of (**A**) AT_1_R, (**B**) gp91p^phox^ or (**C**) SOD2 (green fluorescence) in cells that were stained positively for a neuronal marker, neuron-specific nuclear protein (NeuN) (red fluorescence) in RVLM of ND or HFD offspring at age of 12 weeks. Scale bar: 50 μm.


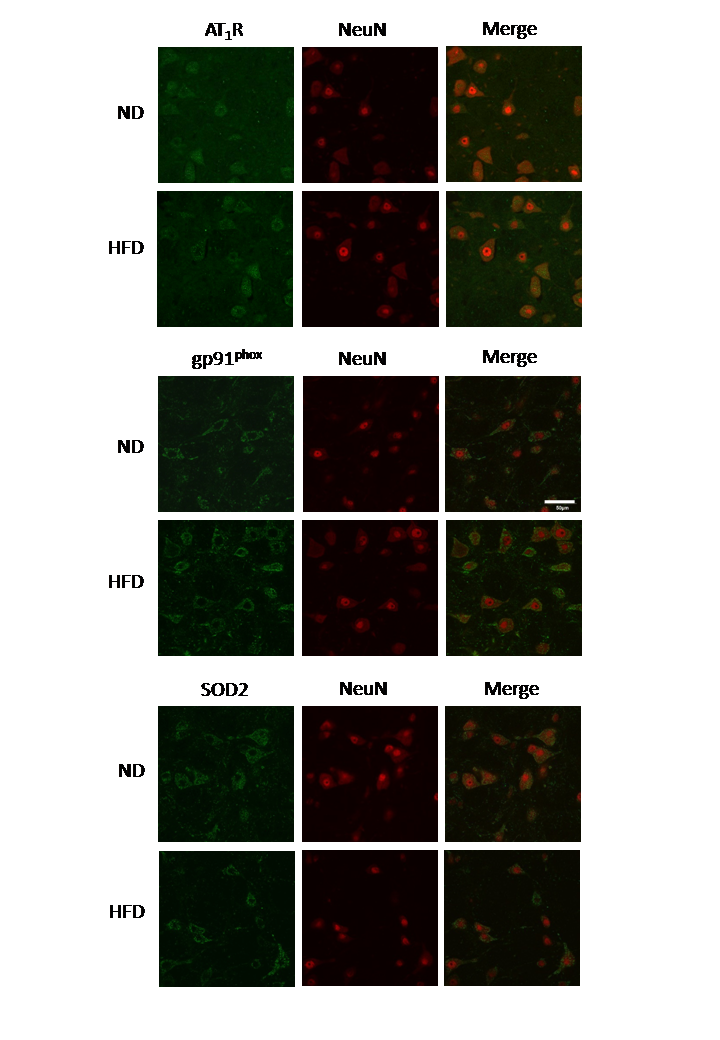


**Figure S2.** Representative gels (insets) and densitometric analysis of results from Western blot analysis showing changes in protein expression of (**A**) p47^phox^ or p67^phox^ of the NADPH oxidase, (**B**) SOD1 or SOD3, (**C**) catalase or GPx, or (**D**) NOS1-3 in RVLM of ND (n = 6-10) or HFD (n = 6-10) offspring, alone or with additional treatment with a HMG-CoA inhibitor, simvastatin (5 mg⋅kg^-1^⋅day^-1^), administered via gastric gavage at age of 8 weeks for 4 weeks. Analysis was performed on tissues collected bilaterally from individual RVLM at age of 12 weeks. Data on protein expression were normalized to the average ND control value, which is set to 1.0, and are presented as mean ± SEM. No significance difference among all groups in one-way ANOVA.


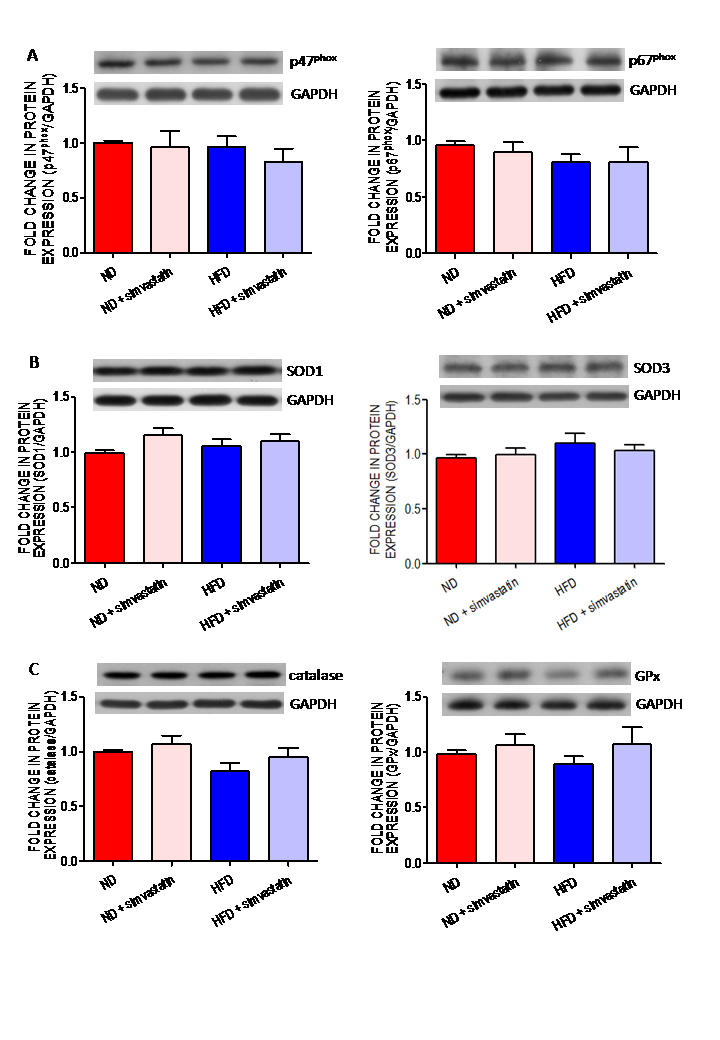


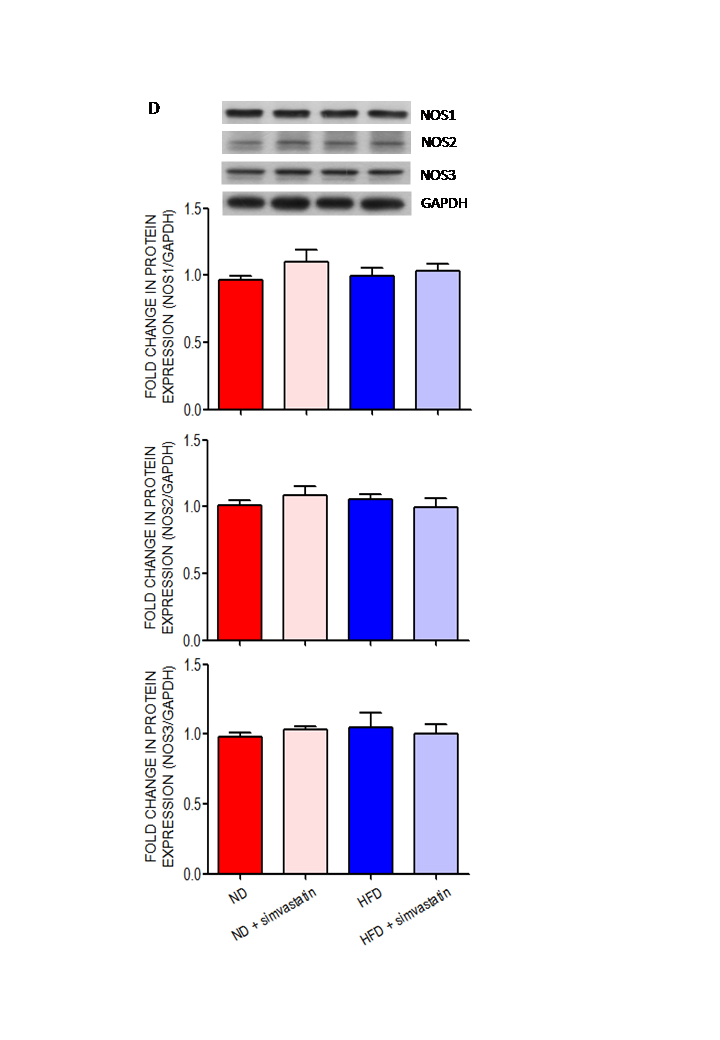


**Figure S3.** Representative gels (insets) and densitometric analysis of results from Western blot analysis showing changes in protein expression of (**A**) p-AMPK and (**B**) SIRT1, as well as (**C**) ratio between p-AMPK/t-AMPK in RVLM of ND or HFD offspring, alone or with additional treatment with an AT1R antagonist, losartan (3 μg⋅μL^-1^⋅h^-1^), microinfused via a minipump into the cistern magna at age of 8 weeks for 4 weeks. Analysis was performed on tissues collected bilaterally from individual RVLM at age of 12 weeks. Data on protein expression were normalized to the average ND control value, which is set to 1.0, and are presented as mean ± SEM (n = 10 in all groups). **P*<0.05 versus ND group in post hoc Newman-Keuls multiple-range test. Group data of p-AMPK and SIRT1 in ND and HFD offspring from Figure 4 are adopted for statistical comparison.


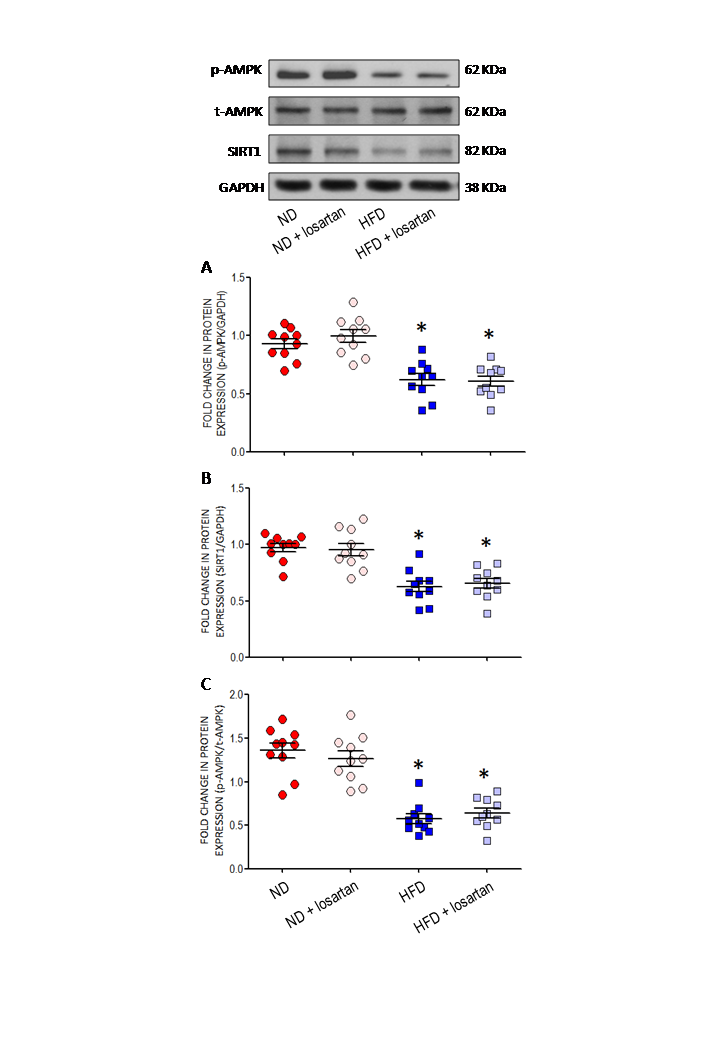

Supplement: Supplementary file 2 — Additional file 2: Figure S1. Representative laser-scanning confocal microscopic images showing the distribution of (A) AT1R, (B) gp91pphox or (C) SOD2 (green fluorescence) in cells that were stained positively for a neuronal marker, neuron-specific nuclear protein (NeuN) (red fluorescence) in RVLM of ND or HFD offspring at age of 12 weeks. Scale bar: 50 μm. Figure S2. Representative gels (insets) and densitometric analysis of results from Western blot analysis showing changes in protein expression of (A) p47phox or p67phox of the NADPH oxidase, (B) SOD1 or SOD3, (C) catalase or GPx, or (D) NOS1–3 in RVLM of ND (n = 6–10) or HFD (n = 6–10) offspring, alone or with additional treatment with a HMG-CoA inhibitor, simvastatin (5 mg·kg− 1·day− 1), administered via gastric gavage at age of 8 weeks for 4 weeks. Analysis was performed on tissues collected bilaterally from individual RVLM at age of 12 weeks. Data on protein expression were normalized to the average ND control value, which is set to 1.0, and are presented as mean ± SEM. No significance difference among all groups in one-way ANOVA. Figure S3. Representative gels (insets) and densitometric analysis of results from Western blot analysis showing changes in protein expression of (A) p-AMPK and (B) SIRT1, as well as (C) ratio between p-AMPK/t-AMPK in RVLM of ND or HFD offspring, alone or with additional treatment with an AT1R antagonist, losartan (3 μg·μL− 1·h− 1), microinfused via a minipump into the cistern magna at age of 8 weeks for 4 weeks. Analysis was performed on tissues collected bilaterally from individual RVLM at age of 12 weeks. Data on protein expression were normalized to the average ND control value, which is set to 1.0, and are presented as mean ± SEM (n = 10 in all groups). *P < 0.05 versus ND group in post hoc Newman-Keuls multiple-range test. Group data of p-AMPK and SIRT1 in ND and HFD offspring from Fig. 4 are adopted for statistical comparison. [file 12929_2020_660_MOESM2_ESM.docx]
